# Identification of multiple arbovirus infections/exposure in Northeastern Brazil using a multiplex microsphere immunoassay

**DOI:** 10.1128/jcm.00013-26

**Published:** 2026-04-30

**Authors:** Wei-Kung Wang, Eduardo Martins Netto, Szu-Chia Hsieh, Guan-Hua Chen, Melissa Barreto Falcão, Celia Pedroso, Rafaela Mayoral, Roger Costa, Yu-Ching Dai, Rennsilve C. Salomon, Jih-Jin Tsai, Laíse de Moraes, Viviane Sampaio Boaventura, Ricardo Khouri, Carlos Brites

**Affiliations:** 1 Department of Tropical Medicine, Medical Microbiology and Pharmacology, John A. Burns School of Medicine, University of Hawaii at Manoa 53738 , Honolulu, Hawaii, USA; 2 Laboratório de Pesquisa em Infectologia (LAPI)-School of Medicine, Universidade Federal da Bahia 28111, Salvador, Brazil; 3 Fundação José Silveira 92924 , Salvador, Bahia, Brazil; 4 Universidade Estadual de Feira de Santana 67836, Feira de Santana, Bahia, Brazil; 5 Tropical Medicine Center, Kaohsiung Medical University Hospital, Kaohsiung, Taiwan; 6 Division of Infectious Diseases, Department of Internal Medicine, Kaohsiung Medical University Hospital 89234, Kaohsiung, Taiwan; 7 School of Medicine, College of Medicine, Kaohsiung Medical University 38023, Kaohsiung, Taiwan; 8 Instituto Gonçalo Moniz –Oswaldo Cruz Foundation (FIOCRUZ), Bahia, Brazil; Mayo Clinic Minnesota, Rochester, Minnesota, USA

**Keywords:** arbovirus, dengue virus, *Zika* virus, *Chikungunya *virus, serosurveillance

## Abstract

Several mosquito-borne arboviruses, including dengue (DENV), Zika (ZIKV), West Nile (WNV), yellow fever (YFV), and chikungunya (CHIKV) viruses, have caused disease outbreaks of public health concern in the tropics and subtropics. The overlap distribution of these arboviruses and cross-reactivities of antibodies to orthoflavivirus envelope protein underscore the need for a reliable and convenient serological test to distinguish these arboviruses in endemic countries. We developed a multiplex IgG microsphere immunoassay (MIA) using 13 antigens to test 8 panels of samples with well-documented arbovirus infections or vaccination (*n* = 374). We further employed this assay, combined with previously reported neutralization test and Western blot analysis to test serum samples (*n* = 300) collected from Saúde, a town in the Northeastern state of Bahia, Brazil. The sensitivity/specificity of mixed DENV1–4, ZIKV, WNV, and YFV nonstructural protein 1 (NS1), and CHIKV virus-like particles (VLP) IgG MIAs were 96.0%/98.3%, 100%/79.9%, 94.4%/73.4%, 52.2%/94.4%, and 100%/99.7%, respectively. The overall low specificity of ZIKV and WNV NS1 was due to cross-reactivities from panels with repeated orthoflavivirus infections, including secondary DENV and DENV + ZIKV infections. In Saúde, we found seropositivity of 70.3%, 22.3%, 39.7%, and 5.7% to DENV, ZIKV, YFV, and CHIKV, respectively, and the majority with multiple arbovirus infections or exposure including DENV + CHIKV, DENV + ZIKV, DENV + YFV, DENV + ZIKV + YFV, and/or CHIKV. Our findings highlight the importance of confirmatory tests for the NS1-based multiplex IgG MIA. This high-throughput assay, combined with confirmatory tests, provides a labor-saving and validated tool for serodiagnosis and serosurveillance of pathogenic arbovirus infections/exposure in endemic regions.

**IMPORTANCE:**

Several multiplex microsphere immunoassays (MIA) have been reported to detect pathogenic arbovirus infections, including dengue (DENV), Zika (ZIKV), West Nile (WNV), yellow fever (YFV), and chikungunya (CHIKV) viruses. None of them included sufficient control panels to validate the sensitivity/specificity of each antigen, confirmatory tests for equivocal samples, or comparison with available commercial kits to advance this technology from research to practical use. We employed eight panels of samples with known arbovirus infections or vaccination (*n* = 374) to evaluate the sensitivity/specificity of our multiplex IgG MIA. We reported high sensitivity of nonstructural protein 1 (NS1)-based IgG MIA (94.4%–100% except YFV NS1) and overall low specificity of ZIKV and WNV NS1 due to cross-reactivities from repeated orthoflavivirus infections, including secondary DENV and DENV + ZIKV infections. Using our algorithm and confirmatory tests, we reported multiple arbovirus infections or exposure (DENV + ZIKV, DENV + YFV, DENV + ZIKV + YFV, and/or CHIKV) in the majority of participants from a town in Northeastern Brazil.

## INTRODUCTION

In the past few decades, several mosquito-borne arboviruses have resulted in disease outbreaks of global health threats in the tropics and subtropics (1). Among these, the four serotypes of dengue virus (DENV1−4), Zika virus (ZIKV), West Nile virus (WNV), and yellow fever virus (YFV) are members of the *Orthoflavivirus* genus of the *Flaviviridae* family, whereas Chikungunya (CHIKV) virus belongs to the *Alphavirus* genus of the *Togaviridae* family (1, 2).

DENV continues to be the leading cause of arbovirus infection and disease in humans, with a historically high number, >14 million reported cases, in 2024 (3–5). Despite three tetravalent live-attenuated DENV vaccines having been tested in phase 3 clinical trials, developing a safe and effective DENV vaccine remains a challenge (6). YFV is endemic in sub-Saharan Africa and tropical America, with ~200,000 severe cases and ~60,000 deaths per year (7). The recent outbreaks in Angola, the Democratic Republic of Congo, Brazil, and Nigeria suggest that YFV has expanded and affected large populations in South America and Africa (7, 8). WNV has caused outbreaks in Africa and Europe and spread to the United States, Canada, and Mexico since 1999 (1, 9). The reports of increased geographic distribution of WNV and travel-related WNV cases highlighted the new challenge of serosurveillance for orthoflaviviruses (1, 9). Since its discovery in 1947, ZIKV was associated with relatively few human cases until the outbreaks on Yap Island in 2007 and in French Polynesia in 2013−2014. This was followed by an explosive spread in the Americas (10); the association of ZIKV with microcephaly and other birth defects has raised global health concerns (11).

CHIKV includes three major lineages: East-Central-South African (ECSA), West African, and Asian lineages (12, 13). The spread of the ECSA lineage to the Indian Ocean, together with the expansion of the Asian lineage, led to its spread in the Americas with ~3.7 million suspected and confirmed cases during 2013–2023, with autochthonous transmission in 114 countries over the tropics and subtropics (12, 13).

Several arboviruses share the same mosquito vectors, such as *Aedes aegypti* and *Aedes albopictus* for DENV, ZIKV, YFV, and CHIKV, whereas WNV is transmitted by the *Culex* mosquito (1, 2). Due to the presence of these mosquitoes in the tropics and subtropics, there is considerable geographic overlap of different arboviruses, such as DENV, ZIKV, and CHIKV in Southeast Asia; DENV, ZIKV, YFV, and CHIKV in Central and South Americas; and DENV, ZIKV, YFV, WNV, and CHIKV in sub-Saharan Africa (1, 2). Clinically, they presented with acute febrile illness and similar symptoms, making them difficult to distinguish (3, 12). Thus, triplex reverse-transcription-polymerase-chain reaction (RT-PCR) tests for DENV, ZIKV, and CHIKV have been used in the endemic regions (14). Due to a short viremia during the acute stage and asymptomatic infection in most individuals, serological tests remain an important component in arbovirus diagnosis and surveillance (1, 14). However, serological cross-reactivities between orthoflaviviruses and between some alphaviruses pose a challenge (1, 14). For orthoflaviviruses, traditional envelope (E) protein-based assays cannot distinguish different orthoflavivirus infections due to the presence of highly conserved fusion loop (FL) residues of the E protein (15–17). According to the guidelines of the Centers for Disease Control and Prevention (CDC), positive or equivocal results of the E protein-based IgM tests require further testing with time-consuming plaque reduction neutralization tests (14). Several serological tests based on orthoflavivirus nonstructural protein 1 (NS1), recombinant E protein domain III, and FL-mutant E protein or virus-like particles (VLP) have shown improved specificity (18–23).

Given its high-throughput and multiplex capacity (up to 100-plex), microsphere immunoassay (MIA) has been employed to detect cytokines, transfusion and transplantation antigens, bacterial and viral infections (24–29). Developing high-throughput and multiplex serological tests of arboviruses is critical for serosurveillance and seroprevalence. Despite several multiplex IgG MIA having been reported to detect pathogenic arbovirus infections, none of them included well-documented control panels to address the sensitivity/specificity of each antigen, or confirmatory tests for equivocal samples from the field. Moreover, the performance of MIA in comparison with available commercial kits remains unclear, making the transition of this technology to practical application challenging. We recently reported that anti-premembrane (prM) antibodies detected by Western blot (WB) analysis can distinguish four orthoflaviviruses (DENV, ZIKV, WNV, and YFV) infection/exposure with a sensitivity/specificity of 88.9%–91.7%/92.5%–99.2%, providing a convenient tool to confirm specific orthoflavivirus infections (30). The objectives of this study were to develop a multiplex IgG MIA for five arboviruses (DENV, ZIKV, WNV, YFV, and CHIKV) based on recombinant proteins and VLP using samples with known arbovirus infections/exposures to determine the sensitivity/specificity, explore its application to samples from endemic areas, and compare its performance with commercial kits.

## MATERIALS AND METHODS

### Human samples

Table S1 summarizes the numbers, sampling time, sources, and confirmation methods of different panels of serum or plasma samples with known arbovirus infections or vaccination. Samples from RT-PCR-confirmed DENV cases were collected between 2006 and 2008 from the Pediatric Dengue Hospital-based Study in Managua, Nicaragua (20), which was approved by the IRBs of the University of California, Berkeley, and the Nicaraguan Ministry of Health. Samples from a ZIKV study in Salvador, Brazil, were confirmed by microneutralization test (NT) as primary ZIKV (pZIKV) (*n* = 13) and previous DENV and ZIKV (DENV + ZIKV) infection (*n* = 22) panels as described previously (31). A total of 300 participants were enrolled with informed consents from Saúde, a municipality with a population of ~11,000 in Bahia, a Northeastern state in Brazil, between June 2021 and February 2022. All residents were invited by the local government to visit study sites at health centers or schools for DENV, ZIKV, and CHIKV serological testing. To ensure coverage of all six areas within Saúde, house-to-house visits were conducted to recruit additional participants in areas where less than 50 participants were enrolled initially. Questionnaires, including basic demographic information and blood sampling, were obtained. The study was approved by the Comitê de Ética em Pesquisa da Maternidade Climério de Oliveira/UFBA, Brazil (CAAE: 25336819.3. 0000.5543/4.691.233, 2019). Samples that tested positive for WNV transcription-mediated amplification (TMA), IgM and IgG antibodies, designated as the WNV infection panel (*n* = 18), were collected from blood donors at the American Red Cross in Gaithersburg, Maryland (20). Samples from a DENV seroprevalence study in Kaohsiung, Taiwan, were confirmed by NT as primary DENV (pDENV), secondary DENV (sDENV) infection, or DENV-negative panel (32, 33); the sampling time was based on questionnaires from study participants. Of the five arboviruses studied, DENV is the only one prevalent in Taiwan (32); thus, the DENV-negative panel from Taiwan is considered a negative serum control for arboviruses in this study. Samples of confirmed CHIKV cases (*n* = 22) were described previously (34). Samples of YFV-17D vaccinees were from the United States (*n* = 10) and Brazil (*n* = 9) based on a history of YF-17D vaccination (30, 31); samples from non-human primates (NHP) receiving YF-17D vaccine (*n* = 4) were from the BEI Resources (NIAID, NIH).

### Recombinant proteins and VLP

The NS1 gene (amino acid residues 1–352) of ZIKV (HPF2013 strain) with a His-tag at the C-terminus and DENV1 (Hawaii strain) E gene (ectodomain: amino acid residues 1–400) were codon-optimized (Integrated DNA Technologies, Skokie, IL) and cloned into the pMT-Bip vector to establish Drosophila S2-cell stable clones (20). ZIKV-NS1 and DENV1 E proteins from supernatants of the stable clones were purified by a fast purification chromatography system (AKTA Pure, GE Health Care Bio-Science, Pittsburg, PA) (20). NS1 proteins of DENV1 (Nauru/Western Pacific/1974), DENV2 (Thailand/16,681/84), DENV3 (Sri Lanka D3/H/IMTSSA-SRI/2000/1266), DENV4 (Dominica/814,669/1981), WNV (NY99), and YFV (17D) were purchased from The Native Antigen (Oxford, UK). CHIKV (H20235-St Martin-2013 strain) VLP was generated by transfection of 293T cells with the CHIKV CE3E2E1 plasmid. DENV1 (Hawaii strain) and mock VLP were generated by transfection of 293T cells with DENV1 wild type (WT) or FL-mutant prME plasmid and mock plasmid, respectively, followed by purification through 20% sucrose cushion ultracentrifugation and quantification by Bradford reagent (MilliporeSigma, Burlington, MA) as described previously (23).

### Coupling of microspheres

Twenty-five micrograms each of recombinant NS1 proteins (DENV1, 2, 3, and 4, mixed DENV1–4, WNV, YFV, and ZIKV) and E protein (DENV1), as well as bovine serum albumin (BSA) and PBS (both negative antigen controls) were coupled onto 5.0 × 10^6^ magnetic carboxylated microspheres (MagPlexTM-C) (Luminex, TX, Austin) using a two-step carbodiimide process at room temperature (35). Mixed DENV1–4 NS1 refers to NS1 proteins from DENV1–4 conjugated on the same bead, whereas individual DENV1, 2, 3, and 4 NS1 indicates NS1 protein from each serotype conjugated to a separate bead. Forty micrograms of WT VLP, FL-mutant VLP (designated as FL VLP), CHIKV VLP, and mock VLP (another negative antigen control) were coupled onto 5.0 × 10^6^ magnetic carboxylated microspheres using the AMG Metallic Cage System (AnteoTech, Australia). The antigen-conjugated microspheres were stored in 400 µL PBN buffer (PBS with 1% BSA and 0.05% sodium azide, Sigma-Aldrich) at 4°C until use.

### MIA

Twenty-five microliters of PBS-1% BSA containing ~1,250 antigen-conjugated microspheres were added to a flat-bottom 96-well plate and incubated with 25 µL diluted serum or plasma (final 1:100 dilution in PBS-1% BSA) at 37°C for 30 min in the dark, followed by wash with 200 µL of PBS-1% BSA twice, incubation with 50 µL of red phycoerythrin-conjugated anti-human or anti-mouse IgG (Jackson Immune Research Laboratory, West Grove, PA) at 37°C for 45 min in the dark, and wash with 200 µL of PBS-1% BSA twice (35). Microspheres were then resuspended in 100 µL of PBS-1% BSA, incubated for 5 min, and read by Magpix (Austin, TX). All incubations were performed on a plate shaker at 700 rpm, and all wash steps used a 96-well magnetic plate separator (MilliporeSigma, Burlington, MA) (35). Each plate includes two positive sera controls (mixed sera from samples with confirmed DENV, ZIKV, YFV, WNV, and CHIKV infections/exposure), four negative sera controls (from the DENV-negative panel), and tested samples (all in duplicates). The MFI was determined for 50 microspheres for each well. The median fluorescence intensity (MFI) values for each antigen were divided by the mean MFI value of one positive serum control (MFI~10^4^) and multiplied by 10^4^ (except by 100 and 3,000 for negative antigen controls and CHIKV VLPs, respectively) to calculate the relative median fluorescence intensity (rMFI) for comparison between plates (Fig. S1). The cutoff rMFI for each antigen was defined as the mean rMFI value of 20 negative serum controls (from the DENV-negative panel) plus 5 standard deviations, which gave a confidence level greater than 99.0% from 4 negatives (36). Each cutoff rMFI was further tested by the receiver-operating characteristics (ROC) analysis and adjusted accordingly (GraphPad Prism 6).

### Pseudovirus NT

Pseudoviruses of CHIKV and Mayaro virus (MAYV), another alphavirus of the same serocomplex as CHIKV, were generated as described previously (37, 38). Briefly, HEK-293T cells, seeded in a 10-cm dish 1 day earlier, were co-transfected with pNL4-3 R-E-miRFP (12 µg) and plasmids CHIKV (12 µg) or MAYV (4 µg) using lipofectamine 2000 and incubated with DMEM media containing 10% FBS (38). The supernatants were collected at 72 h post-transfection, followed by low-speed centrifugation at 300 × *g* for 10 min, aliquoted, titrated, and stored at −80°C (38). For NT, Huh7 cells, seeded onto 96-well plates (2 × 10^4^ cells/well) one day earlier, were inoculated with mixtures containing pseudovirus (CHIKV or MAYV) and fourfold serial dilutions of plasma (1:1) for spin infection (centrifuged at 1,200 × *g* at 4°C for 1 h and followed by incubation at 37°C for 1 h); the plates were scanned at 72 h by Li-Cor Odyssey imager (Fig. S2A and B) (38). The % of infection at different plasma dilutions (from 1:10 to 1:10,240 dilutions) was calculated by the formula (intensity of serum + pseudovirus – intensity of media only)/(intensity of pseudovirus only – intensity of media only) × 100. The % neutralization = 100 – % of infection (38). NT_50_ titer was the plasma dilution that reached 50% neutralization using 4-parameter nonlinear regression analysis (GraphPad 6.0, Boston, MA). NT_50_ titer < 10 was arbitrarily assigned as 5.

### Euroimmun ELISA kits

Euroimmun anti-DENV, ZIKV, and CHIKV IgG ELISA kits, which detect antibodies against DENV particles (mainly E protein), ZIKV NS1 protein, and recombinant CHIKV antigen, respectively, were used to test samples collected in Saúde, Bahia, Brazil.

### Statistical analysis

Two-tailed Mann-Whitney test and Fisher’s exact test were used to compare quantitative and qualitative variables, respectively, between two groups (GraphPad Prism 6). The 95% confidence interval (CI) was calculated using Excel. The positive, negative, and overall agreements and kappa assessment were calculated using the SPSS 20.

## RESULTS

### Establishing an arbovirus multiplex IgG MIA

We first employed an IgG MIA containing 15 beads, including 12 antigens: NS1 proteins of DENV1, 2, 3, and 4, mixed DENV1–4, ZIKV, WNV, and YFV, E protein of DENV1, VLP of CHIKV, and DENV1 (WT and FL VLP), and three negative antigen controls (PBS, BSA, mock VLP) to test 374 serum or plasma samples from 8 panels with known arbovirus infections/vaccination (Table 1). For orthoflaviviruses, DENV NS1 proteins (either mixed DENV1−4 or each serotype) were recognized primarily by DENV panels (pDENV, sDENV, and DENV + ZIKV) rather than non-DENV panels, including negative sera, pZIKV, WNV, and YFV-17D panels (Table 1). ZIKV NS1 was recognized by ZIKV panels (pZIKV, DENV + ZIKV) and 54.1% (60/111) of the sDENV panel. WNV NS1 was recognized by the WNV panel as well as the sDENV and DENV + ZIKV panels, suggesting cross-reactivity to WNV NS1 after repeated orthoflavivirus infections, whereas YFV NS1 was recognized mainly by the YFV-17D panel. DENV1 VLP (WT or FL) was recognized by most orthoflavivirus samples tested except the negative sera panel, suggesting they can be used as a pan-orthoflavivirus marker specific to the current multiplex panel (DENV, ZIKV, WNV, and YFV). Further comparison revealed that DENV1 WT VLP had higher sensitivity for the current orthoflavivirus panel than FL VLP (94.5 vs 91.2%) (Table 1), suggesting that DENV1 WT VLP alone can serve as a representative pan-orthoflavivirus marker. For alphavirus, CHIKV VLPs were mainly recognized by the CHIKV panel. As expected, three negative antigen controls were not recognized by any panel (data not shown).

**TABLE 1:** Results of multiplex IgG MIA with 12 antigens for 8 known serum/plasma panels.

No. of positive/total samples (%) in different serum/plasma panels^*a*^								
IgG MIA^*c*^	DENV negative^*d*^	pDENV	sDENV	pZIKV	DENV+ZIKV	WNV^*b*^	YF-17D	CHIKV
D1−4 NS1	1/135 (0.7%)	**36/40 (90.0%)**	**108/111 (97.3%)**	0/12 (0%)	**22/22 (100%)**	1/18 (5.6%)	1/14*^g^* (7.1%)	NA^*h*^
D1 NS1	1/135 (0.7%)	**33/40 (82.5%)**	**111/111 (100%)**	2/12 (16.7%)	**22/22 (100%)**	1/18 (5.6%)	2/14^*g*^ (14.3%)	NA^*h*^
D2 NS1	1/135 (0.7%)	**36/40 (90.0%)**	**108/111 (97.3%)**	0/12 (0%)	**22/22 (100%)**	2/18 (11.1%)	2/14^*g*^ (14.3%)	NA^*h*^
D3 NS1	1/135 (0.7%)	**33/40 (82.5%)**	**107/111 (96.4%)**	2/12 (16.7%)	**22/22 (100%)**	2/18 (11.1%)	2/14^*g*^ (14.3%)	NA^*h*^
D4 NS1	3/135 (2.2%)	**34/40 (85.0%)**	**105/111 (94.6%)**	1/12 (8.3%)	**22/22 (100%)**	1/18 (5.6%)	1/14^*g*^ (7.1%)	NA^*h*^
ZIKV NS1	0/135 (0%)	3/40 (7.5%)	60/111 (54.1%)	**12/12 (100%)**	**22/22 (100%)**	0/18 (0%)	1/14^*g*^ (7.1%)	NA^*h*^
WNV NS1	0/135 (0%)	4/40 (10.0%)	62/111 (55.9%)	1/12 (8.3%)	20/22 (90.9%)	**17/18 (94.4%)**	2/14^*g*^ (14.3%)	NA^*h*^
YFV NS1	0/135 (0%)	0/40 (0%)	11/111 (9.9 %)	NA^*e*^	NA^*e*^	NA^*e*^	**12/23 (52.2%)**	NA^*h*^
								
D1 WT VLP	0/135 (0%)	**40/40 (100%)**	**111/111 (100%)**	10/12 (83.3%)	**22//22 (100%)**	18/18 (100%)	4/14^*g*^ (28.6%)	NA^*h*^
D1 FL VLP	0/135 (0%)	**40/40 (100%)**	**111/111 (100%)**	6/12 (50.0%)	**22//22 (100%)**	15/18 (83.3%)	4/14^*g*^ (28.6%)	NA^*h*^
D1 E	2/135 (1.5%)	**38/40 (95.0%)**	**110/111 (99.1%)**	8/12 (66.7%)	**22/22 (100%)**	18/18 (100%)	2/14^*g*^ (14.3%)	NA^*h*^
								
CHIKV VLP	0/135 (0%)	0/40 (0%)	0/111 (0%)	NA^*f*^	NA^*f*^	0/18 (0%)	1/23 (4.3%)	**22/22 (100%)**

^*a*^ pDENV, primary DENV infection; sDENV, secondary DENV infection; pZIKV, primary ZIKV infection; DENV + ZIKV, previous DENV and ZIKV infections; WNV, WNV infection; YF-17D, YF-17D vaccination; CHIKV, CHIKV infection.

^*b*^ Index samples tested positive for WNV transcription-mediated amplification, IgM, and IgG from blood donors at the American Red Cross (20).

^*c*^ NS1, nonstructural protein 1; VLP, virus-like particles; E, envelope protein; WT, wild type; FL, fusion loop-mutated; D1, DENV1; D2, DENV2; D3, DENV3; D4, DENV4. No. (%) of homologous NS1, VLP, or E recognized by each panel are bolded.

^*d*^ DENV-negative panel confirmed by microneutralization test is considered a negative sera control for the five arboviruses tested as described in Materials and Methods.

^*e*^ NA, not applicable. Due to the lack of history of YFV vaccination or infection in these panels, the data were not included in the analysis of sensitivity/specificity.

^*f*^ Due to the lack of history of CHIKV infection in these panels, the data were not included in the analysis of sensitivity/specificity.

^*g*^ Due to the lack of history of DENV, ZIKV, or WNV infection from a subset of the YF-17D (*n* = 9) panel from Brazil, these samples were not included in the analysis of DENV, ZIKV, and WNV NS1 or VLP recognition.

^*h*^ Due to the lack of history of DENV, ZIKV, or YFV vaccination or infection of the CHIKV (*n *= 22) panel from Brazil, these samples were not included in the analysis of DENV, ZIKV, WNV, and YFV NS1 or VLP recognition.

**Fig 1:**
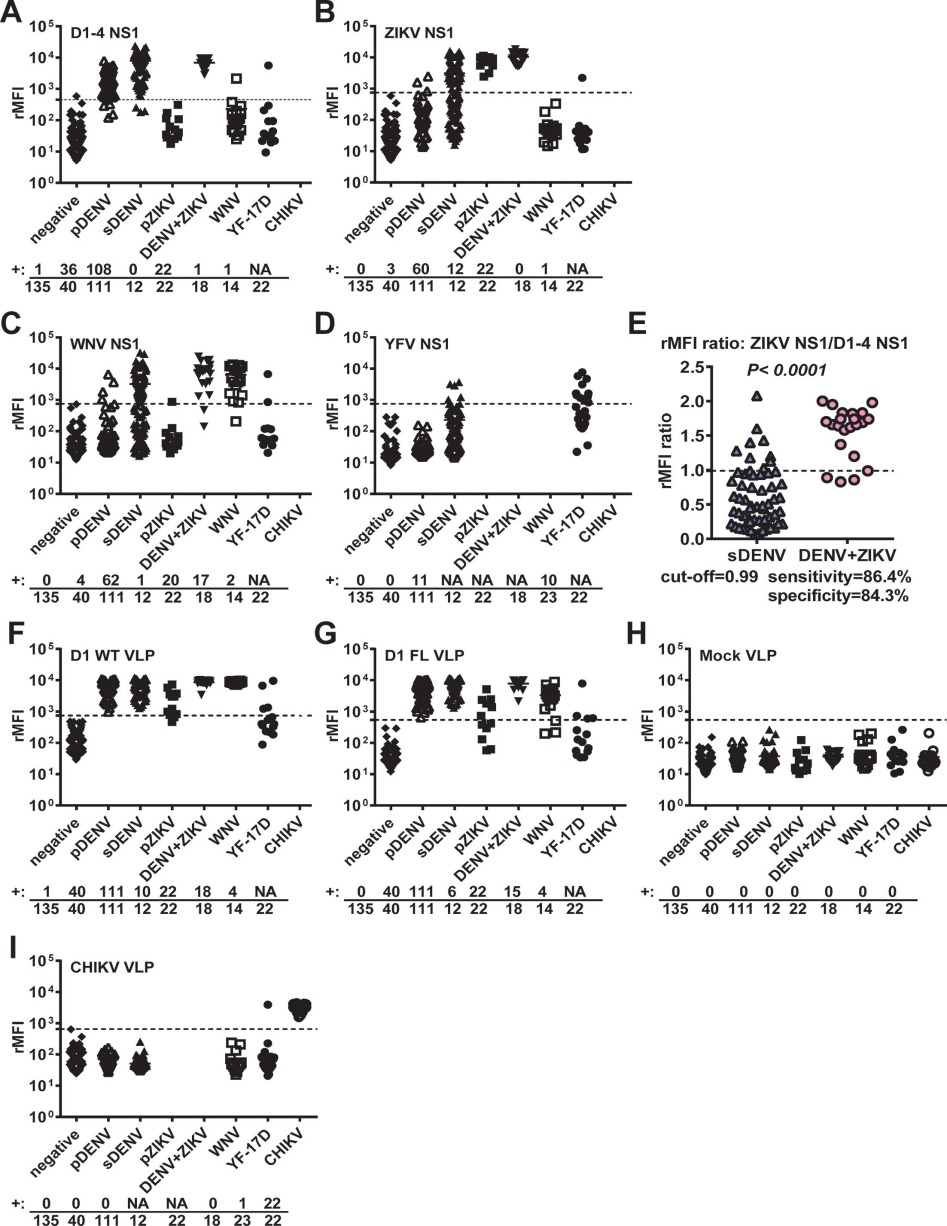
Results of multiplex IgG, including seven down-selected antigens and one negative antigen control. (**A–D and F–I**) Results of IgG MIA, including seven antigens and one negative antigen control: DENV1−4 NS1 (**A**), ZIKV NS1 (**B**), WNV NS1 (**C**), YFV NS1 (**D**), DENV1 WT VLP (**F**) and FL VLP (**G**), mock VLP (**H**), and CHIKV VLP (**I**). The eight panels of serum/plasma samples with known arbovirus infection/exposure are pDENV, sDENV, pZIKV, DENV+ZIKV, and WNV, YFV-17D vaccination, and negative sera panels. The number of positive and total samples tested for each panel is shown below each graph. NA, not applicable due to the lack of history of DENV, ZIKV, CHIKV infection, or YFV infection or vaccination in some panels. (**E**) The ratio of rMFI of ZIKV to DENV1−4 NS1 comparing the sDENV and DENV + ZIKV panels. *P* = 0.0001, two-tailed Mann-Whitney test. Data are the means (each in duplicate), and dashed lines indicate cutoff rMFI.

In agreement with previous reports that anti-DENV NS1 IgG antibodies following pDENV or sDENV infection recognized one to three or all four DENV serotypes, respectively (16, 20), mixed DENV1−4 NS1 had higher sensitivity/specificity (96.0/98.3%) than individual DENV serotype NS1 (Table S2), and were thus chosen for further testing. Additionally, DENV1 WT and FL VLP had higher sensitivity (100%) than recombinant E protein (86.0%) (Table S3) and were chosen for further testing. Thus, we down-selected from 12 to 7 antigens and summarized the results in Fig. S3 and Fig. 1, which was a graphical presentation of the 7 down-selected antigens. To distinguish the sDENV and DENV + ZIKV panels, we found the ratio of rMFI of ZIKV to DENV1−4 NS1 was significantly different between them, with a cutoff ratio of 0.99 (Fig. 1E).

The overall sensitivity/specificity of DENV1−4, ZIKV, WNV, and YFV NS1 IgG MIAs were 96.0/98.3%, 100%/79.9%, 94.4%/73.4%, and 52.2%/94.4%, respectively (Table 2). For alphavirus, the overall sensitivity/specificity for CHIKV VLP was 100/99.7%.

**TABLE 2:** Sensitivity and specificity of different NS1 and VLP IgG MIA.

Viral antigen^*c*^	Group	% Sensitivity (95% CI)^*a*,*b*^	% Specificity (95% CI)^*a*,*b*^
D1−4 NS1	Overall	96.0 (93.0–97.5)	98.3 (96.4–99.3)
	Subgroup	pDENV:90.0, sDENV:97.3, DENV + ZIKV:100	DENV-negative:99.3, pZIKV:100, WNV:94.4, YF-17D:92.9
ZIKV NS1	Overall	100 (100–100)	79.9 (75.5–82.1)
	Subgroup	pZIKV:100, DENV + ZIKV:100	DENV-negative:100, pDENV:92.5, sDENV:46.0, WNV:100, YF-17D:92.9
WNV NS1	Overall	94.4 (83.9–99.8)	73.4 (68.6–75.8)
	Subgroup	WNV:94.4	DENV-negative:100, pDENV:90, sDENV:44.1, pZIKV:91.7, DENV + ZIKV:9.1, YF-17D:85.7
YFV NS1	Overall	52.2 (31.8–62.6)	94.4 (91.7–95.8)
	Subgroup	YF17D:52.2	DENV-negative:100, pDENV:100, sDENV:90.1, pZIKV:NA^*d*^, DENV + ZIKV:NA^*d*^, WNV:NA^*d*^
D1 WT VLP	Overall	100 (100–100)	82.1 (76.5–85.0)
	Subgroup	pDENV:100, sDENV:100, DENV+ZIKV:100	DENV-negative:100, pZIKV:16.7, WNV:0, YF-17D:71.4
D1 FL VLP	Overall	100 (100–100)	86.0 (81.0–88.6)
	Subgroup	pDENV:100, sDENV:100, DENV+ZIKV:100	DENV-negative:100, pZIKV:50.0, WNV:16.7, YF-17D:71.4
CHIKV VLP	Overall	100 (100–100)	99.7 (99.1–100)
	Subgroup	CHIKV:100	DENV-negative:100, pDENV:100, sDENV:100, pZIKV:NA^*e*^, DENV + ZIKV:NA^*e*^, WNV:100, YF-17D:95.7

^*a*^ CI, confidence interval. pDENV, primary DENV infection; sDENV, secondary DENV infection; pZIKV, primary ZIKV infection; DENV + ZIKV, previous DENV and ZIKV infections; WNV, WNV infection; YF-17D, YF-17D vaccination; CHIKV, CHIKV infection.

^*b*^ For simplicity, the 95% CIs in the subgroup are not shown.

^*c*^ NS1, nonstructural protein 1; VLP, virus-like particles; WT, wild type; FL, fusion loop-mutated; D1, DENV1; D2, DENV2; D3, DENV3, D4, DENV4.

^*d*^ NA, not applicable; the history of YFV vaccination or infection was not available in these panels.

^*e*^ NA, not applicable; the history of CHIKV infection was not available in these panel.

### Cross-reactivity to multiple NS1 proteins after sDENV infection

**Fig 2:**
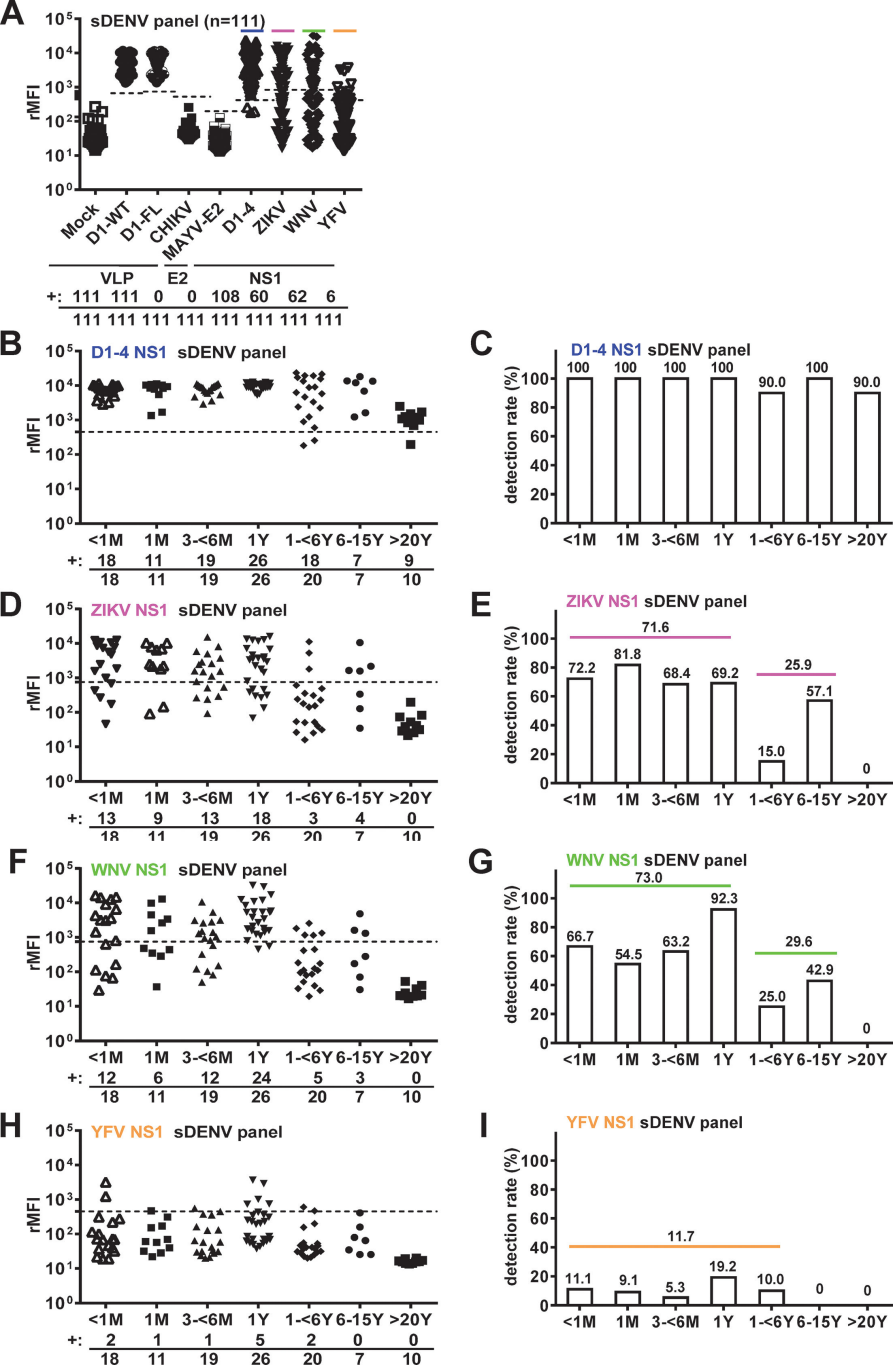
Results of NS1 IgG MIA and cross-reactivities over time after sDENV infection. Results of multiplex IgG MIA in sDENV panel (**A**), and NS1 IgG MIA and detection rate over time, including DENV1−4 (**B and C**), ZIKV (**D and E**), WNV (**F and G**), and YFV (**H and I**) NS1 proteins. Numbers of positive and total samples tested for each subgroup are shown below each graph with rMFI. Cross-reactivities (%) to ZIKV, WNV, and YFV NS1 within 1 or 6 years or between 1 and 15 years are shown above the lines in each graph (**E, G, and I**). Data are the means (each in duplicate), and dashed lines indicate cutoff rMFI.

To investigate NS1 cross-reactivity following repeated orthoflavivirus infections, we analyzed the sDENV panel (Fig. 2A), which included 111 samples with known sampling time post-symptom onset (PSO). The detection rates for DENV1−4 NS1 were 100% up to 1 year and 90%–100% after 1–20 years PSO (Fig. 2B and C). The cross-reactivity to ZIKV NS1 decreased from 71.6% (within 1 year) to 25.9% (1–15 years) (Fig. 2D and E). A similar trend was observed for the cross-reactivity to WNV NS1 (Fig. 2F and G). The cross-reactivity to YFV NS1 was lower (11.7% up to 6 years PSO). As a comparison, we further analyzed anti-NS1 antibodies after pDENV infection; the detection rates for DENV1−4 NS1 remained 100% up to 6 years and declined to 42.9% >20 years (Fig. S4A through C). The cross-reactivity to ZIKV and WNV NS1 was lower, 7.5% and 10%, respectively, mainly within 1 year (Fig. S4D through G); no cross-reactivity to YFV NS1 was observed.

### Proposed algorithm for determining orthoflavivirus and alphavirus infections

**Fig 3:**
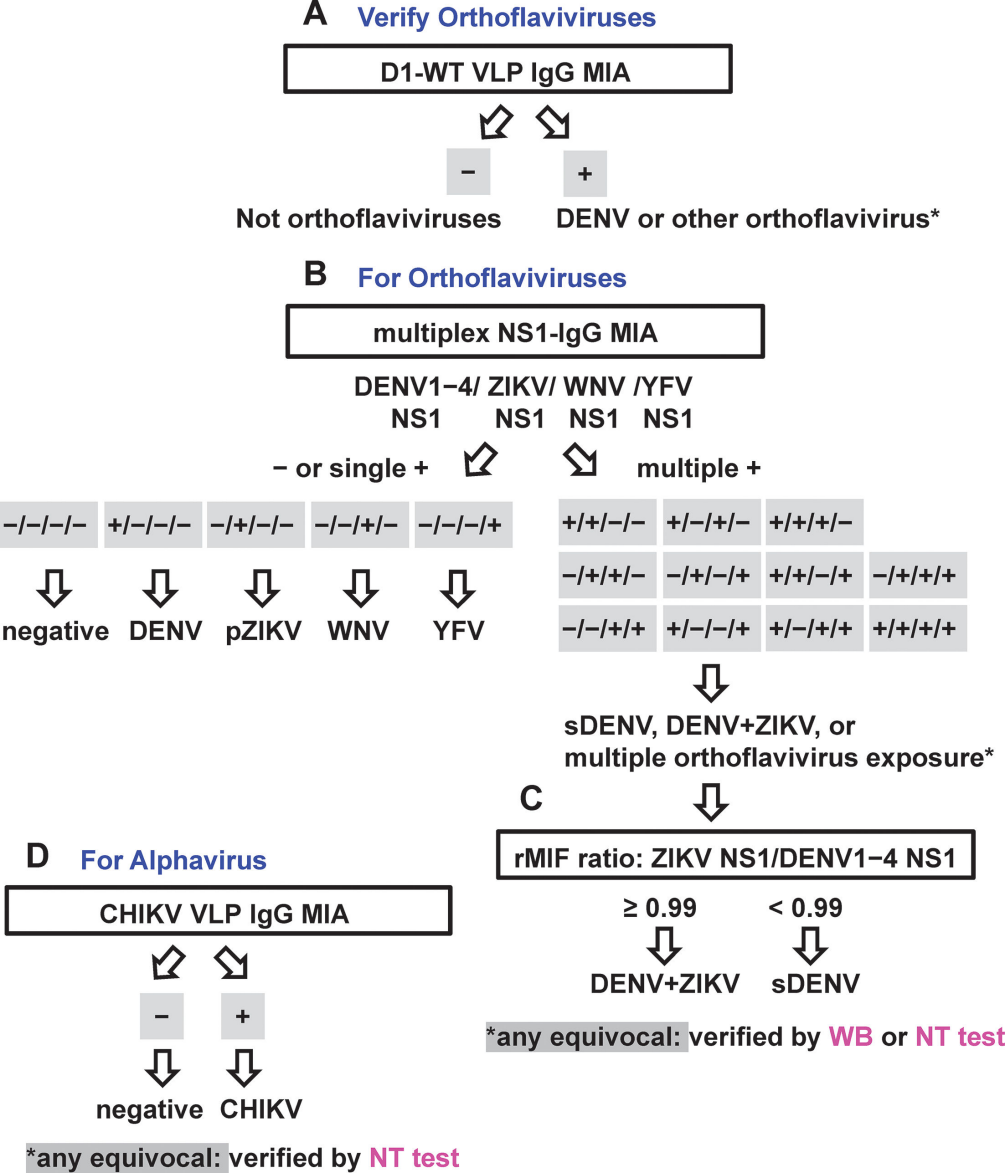
Proposed algorithm of using multiplex IgG MIA to determine orthoflavivirus and alphavirus infections. (**A**) Samples that tested negative or positive to DENV1 WT VLP represent no or infections/exposure to orthoflaviviruses, respectively. (**B**) For orthoflaviviruses, based on positivity to NS1 antigens of four serocomplexes (DENV1−4, ZIKV, WNV, and YFV), samples that tested negative to all or positive for one of four NS1 antigens represent orthoflavivirus negative or single orthoflavivirus (DENV, pZIKV, WNV, or YFV) infection/exposure, respectively. Samples that tested positive for two or more NS1 antigens represent multiple orthoflavivirus infections/exposure. (**C**) To distinguish between DENV + ZIKV and sDENV infections, the rMFI ratio of ZIKV to DENV1−4 NS1 ≥ or < 0.99 suggests DENV + ZIKV or sDENV infection, respectively. All equivocal samples will be verified by previously reported WB analysis or NT. (**D**) For alphavirus, samples that tested negative or positive to the CHIKV VLP suggest negative or CHIKV infection, respectively. All equivocal samples will be verified by pseudovirus NT (to both CHIKV and MAYV).

As anti-NS1 antibodies are generally more specific to each orthoflavivirus than anti-E antibodies that are cross-reactive and recognize VLP (Table 2), we proposed to use positivity to DENV1 WT VLP to verify the four orthoflaviviruses (DENV, ZIKV, WNV, and YFV) first (Fig. 3A), followed by NS1 positivity to determine different orthoflavivirus infections/exposure. NS1 positivity to one of the four orthoflaviviruses tested indicates single orthoflavivirus infection/exposure (Fig. 3B); multiple NS1 positivities suggest multiple orthoflavivirus infections/exposure, such as sDENV or DENV + ZIKV infection. If these two are suspected, the rMFI ratio of ZIKV to DENV1−4 NS1 ≥ or <0.99 suggests DENV + ZIKV or sDENV infection/exposure, respectively (Fig. 3C). All equivocal samples will be confirmed by specific anti-prM antibodies to DENV, ZIKV, WNV, and YFV in WB analysis, or by NT (30, 31, 33). For alphavirus, positivity to CHIKV VLP suggests CHIKV infection. To rule out the possibility of MAYV infection, another alphavirus present in Central and South America with a similar clinical presentation (38–40), pseudovirus NT is recommended for equivocal samples and confirmation of CHIKV infection (Fig. 3D, Fig. S2B).

### Arbovirus seroprevalence in Saúde

**Fig 4:**
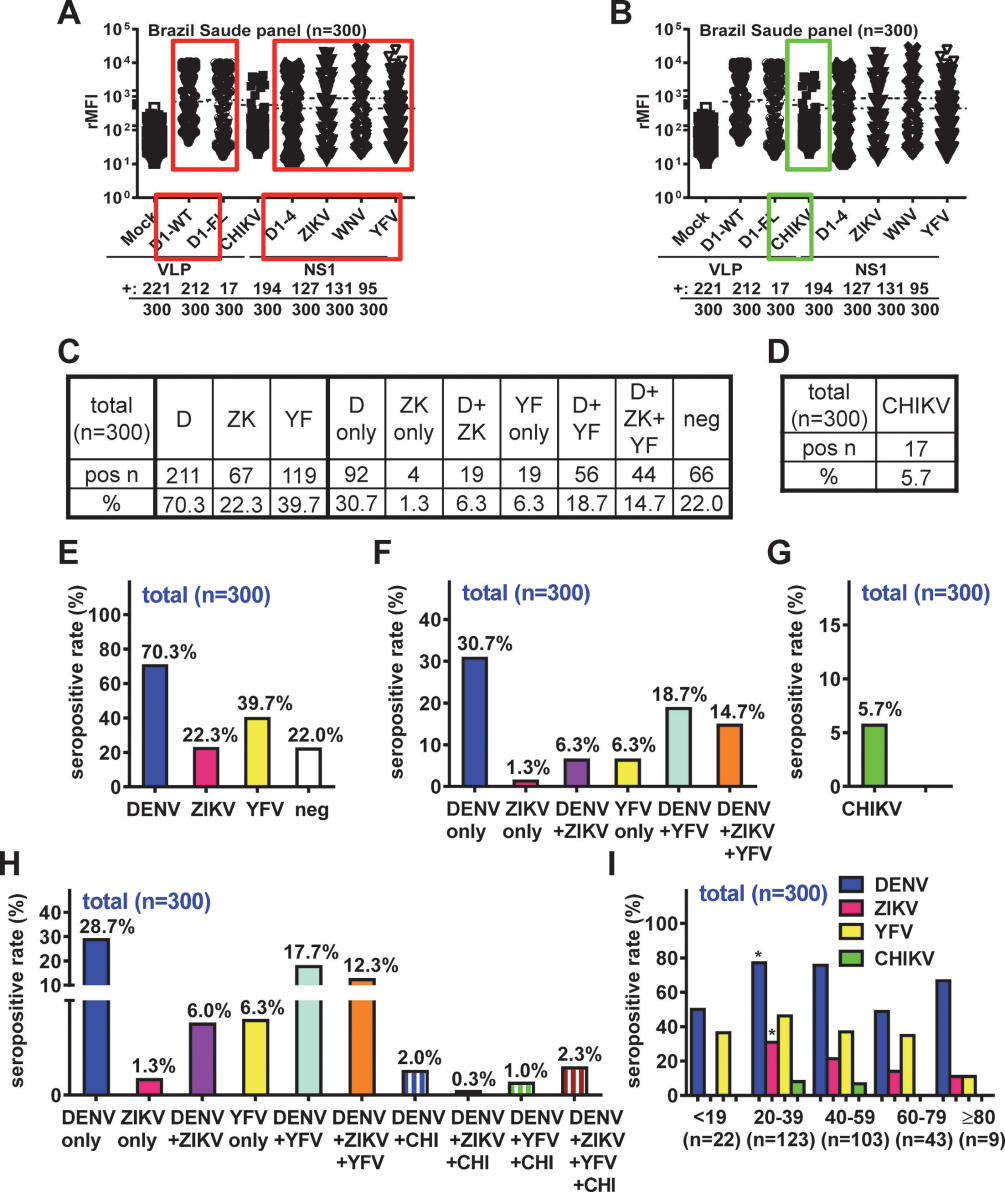
Seroprevalence of orthoflaviviruses and alphavirus in Saúde. (**A and B**) Multiplex IgG MIA tested for 300 serum samples collected from Saúde, with results of four orthoflaviviruses (**A**) and one alphavirus (**B**) highlighted. Numbers of positive and total samples tested for each antigen are shown below. (**C and D**) The number of samples tested positive for one or multiple virus infections and the total number of samples tested for orthoflaviviruses (**C**) and alphavirus (**D**). (**E–H**) Seropositive rates of one or multiple virus infections for orthoflaviviruses (**E and F**), alphavirus (**G**), and combining orthoflaviviruses and alphavirus together (**H**). (**I**) Age-stratified seroprevalence of DENV, ZIKV, YFV, and CHIKV. *P < 0.01, comparing age groups ≤19 and 20–39 for DENV and ZIKV, Fisher’s exact test.

We next tested serum samples collected from participants in Saúde. The age ranged from 11 to 91 years old (mean: 42.1) with the proportion of age groups comparable to that of the population in Saúde (Table S4); the male to female ratio was 1.24. For orthoflaviviruses, we found 193 had DENV infection, 66 ZIKV, 96 YFV, and 66 negative (Fig. S5A and B). Notably, 20 were interpreted as unspecified orthoflavivirus infection based on positivity to DENV1 WT VLP and negative to all NS1 tested (Fig. S5C); after verification by WB analysis, 7 had DENV infection, 12 DENV + YFV, and 1 YFV (Fig. S5D and E). Fifteen were possible WNV infections based on positivity to WNV NS1 (*n* = 131) and exclusion of sDENV (*n* = 57) and DENV + ZIKV (*n* = 59) infections, considering their high cross-reactivity to WNV NS1 (55.9% and 90.9%, respectively) (Table 1, Fig. S5F). After verification by WB analysis, 3 had DENV infection, 11 had DENV + YFV, 1 had YFV (Fig. S5G and H), and none had WNV infection. These findings highlight the importance of confirmatory tests for the NS1-based multiplex IgG MIA. Together, we found a seropositive rate of 70.1% to DENV, 22.3% to ZIKV, 39.7% to YFV, and 22% negative, including multiple orthoflavivirus infections (DENV + ZIKV, DENV + YFV, and DENV + ZIKV + YFV) (Fig. 4A, C, E and F).

For alphavirus, 17 tested positive for the CHIKV VLP (Fig. S6A and B). Consistent with the CHIKV-positive control panel (Fig. S2B), the ratios of NT_50_ titers of CHIKV to MAYV were ≥2.5 for 16 samples tested, which had sufficient volume, confirming CHIKV infection (Fig. S6C). The results of alphavirus infection are summarized in Fig. 4B, D and G. Taken together, we found the majority (53.4%) of positive samples had multiple arbovirus infections or exposure, including DENV + CHIKV, DENV + ZIKV, DENV + YFV, DENV + ZIKV + YFV, and/or CHIKV (Fig. 4H). We further determined the age-stratified seroprevalence of DENV, ZIKV, YFV, and CHIKV and the relationship to sex (Fig. 4I, Table S4).

### Comparison of multiplex IgG MIA and commercial DENV/ZIKV/CHIKV kits

**Fig 5:**
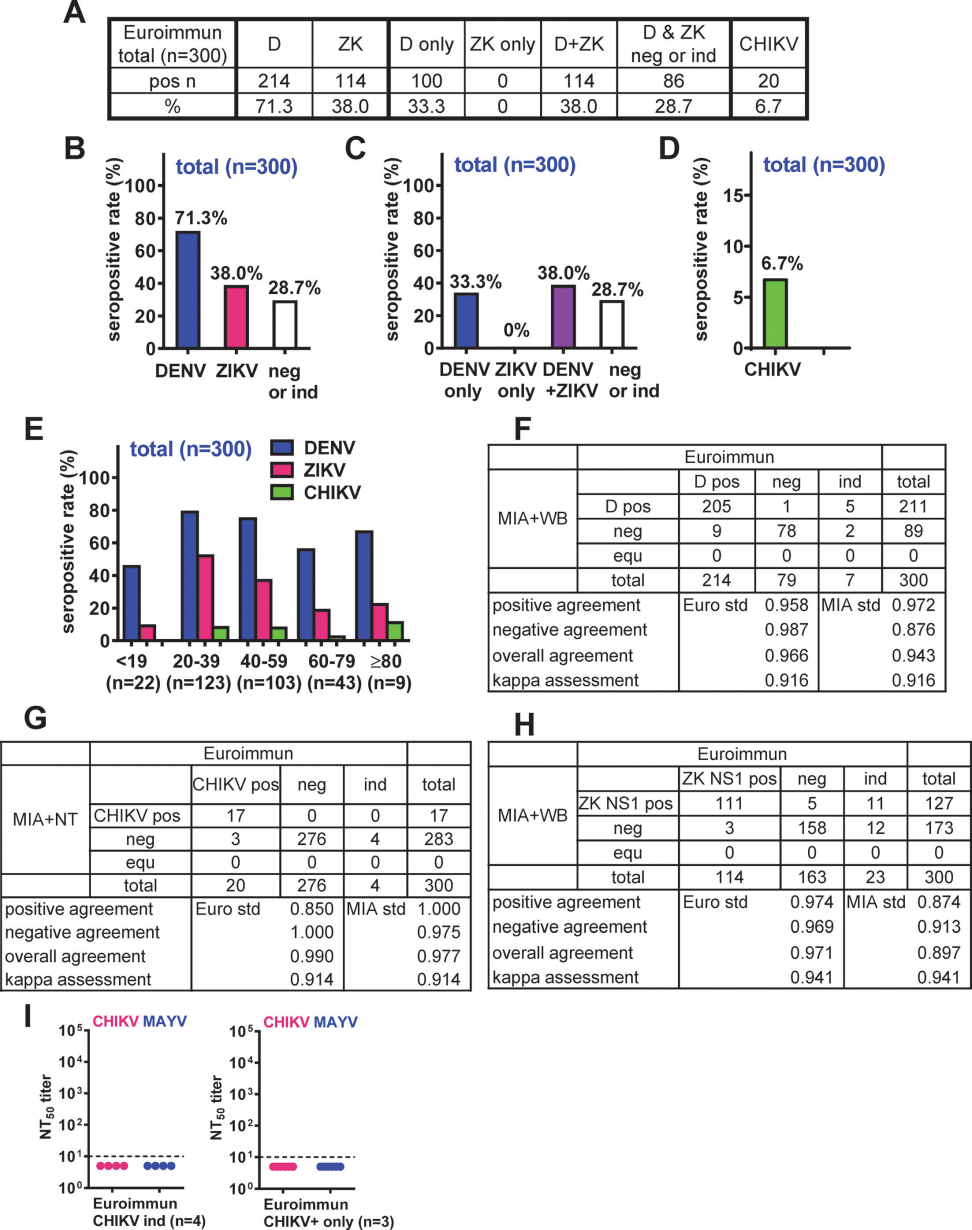
Seroprevalence of DENV, ZIKV, and CHIKV in Saúde based on commercial ELISA kits and comparison with IgG MIA plus confirmatory tests. (**A**) The number of samples tested positive for DENV, ZIKV, DENV only, ZIKV only, DENV + ZIKV, CHIKV, and the total number of samples tested. (B−D) Seropositive rates of DENV and ZIKV (**B**), DENV only, ZIKV only, DENV + ZIKV (**C**), and CHIKV (**D**). (**E**) Age-stratified seroprevalence of DENV, ZIKV, and CHIKV. (F−H) Comparison of the performance of Euroimmun IgG kits and IgG MIA plus confirmatory tests for DENV (**F**), CHIKV (**G**), and ZIKV (**H**). The positive, negative, and overall agreements and kappa assessment of two assays based on either one as the gold standard. pos: positive, neg: negative, ind: indeterminate, and equ: equivocal. (**I**) Results of NT (NT_50_ titers) to CHIKV and MAYV pseudoviruses for samples tested positive or indeterminate for CHIKV by Euroimmun IgG kits only.

We further tested samples with the Euroimmun IgG ELISA kits, which were commonly used in Brazil and other endemic countries. A seropositive rate of 71.3% to DENV, 38.0% to ZIKV, 6.7% to CHIKV, and 28.7% negative were found (Fig. 5A through D), with the age-stratified seroprevalence shown in Fig. 5E. The overall agreements between our multiplex IgG MIA and the Euroimmun ELISA kits for DENV, ZIKV, and CHIKV were 94.3%–96.6%, 89.7%–97.1%, and 97.7%–99.0%, respectively, and the kappa assessments were high (0.914–0.941). (Fig. 5F through H). For the three samples that tested positive and the four intermediates using the Euroimmun CHIKV IgG kit, the NT_50_ titers to CHIKV were <10, excluding CHIKV infection (Fig. 5I).

## DISCUSSION

Since the ZIKV outbreak in 2015−2017, two studies reported that prior DENV infection reduces the risk of symptomatic ZIKV infection, whereas others showed that previous ZIKV or DENV followed by ZIKV infection increased the risk of symptomatic DENV2 and severe disease, while two or more DENV infections with prior ZIKV were protective (41–44). In addition, preexisting Japanese encephalitis virus (JEV) neutralizing antibodies were reported to increase the risk of symptomatic DENV infection, and preexisting DENV IgG was associated with improved JEV disease outcome (45, 46). Other studies reported the effect of YFV exposure on DENV disease outcome and vice versa (47). Together, these studies demonstrate the complexity of interactions between DENV, ZIKV, and/or other orthoflaviviruses, while highlighting the importance of orthoflavivirus infection/exposure histories for understanding the epidemiology, pathogenesis, and vaccine strategies in regions where multiple members co-circulate. In this regard, our multiplex IgG MIA assay in combination with confirmatory tests provides a convenient and validated tool to explore DENV, ZIKV, WNV, CHIKV, and YFV infections/exposure in arbovirus-endemic countries.

The low sensitivity (52.2%) of anti-YFV NS1 IgG for detecting YFV infection/exposure was in agreement with previous reports (44.4%) for YF-17D vaccinees and natural infection (28, 48). It is worth noting that most samples in our YFV control panel were from YF-17D vaccinees without other orthoflavivirus infection, which may contribute to lower anti-YFV antibody titers and lower detection rate of anti-YFV NS1 than those with other orthoflavivirus infection. This was supported by a recent study showing lower neutralizing antibody titers against diverse YFV strains, such as South America genotype I, among YF-17D vaccinees without evidence of heterologous orthoflavivirus infection (49). As previous studies reported genotype-specific differences in anti-E neutralizing antibody titers against the YF-17D strain vs. the South America genotype I virus (50, 51), the possibility that genotype-specific differences in anti-YFV NS1 antibodies may affect the detection rate of anti-YFV NS1 based on the YF-17D vaccine strain should also be considered. Consistent with a recent report, the specificity of our DENV1-4, ZIKV, WNV, and YFV NS1 IgG MIA based on negative sera control was high (99.3%–100%) (Table 2) (29). To reflect the real-world situations, we calculated the overall specificity based on all tested panels that have known arbovirus infections/vaccination (Table 2). The low overall specificity of ZIKV and WNV NS1 proteins was mainly due to cross-reactivities among those with repeated orthoflavivirus infections, including sDENV and DENV + ZIKV panels. Using our sDENV panel with known sampling time, we showed the relationship between sampling time and cross-reactivities to ZIKV or WNV NS1, which were considerably high up to 15 years PSO, despite a declining trend from ~72% (within 1 year) to ~27% (up to 15 years). Notably, the low cross-reactivity observed in the sDENV and DENV + ZIKV panels against YFV NS1 relative to WNV NS1 could be due to lower amino sequence homology between DENV and YFV NS1 (~38.6%–40.9%) compared with that between DENV and WNV NS1 (~50.2%–54.8%) (52). Alternatively, the limited sensitivity of YFV NS1 may account for the comparatively low cross-reactivity observed in the sDENV and DENV + ZIKV panels against YFV NS1 relative to WNV NS1.

In Saúde, we found seropositivity of 70.3%, 22.3%, 39.7%, and 5.7% to DENV, ZIKV, YFV, and CHIKV, respectively. None was found to have WNV infection (Fig. S5F through H), which is consistent with the observation that no WNV infection in humans has been reported in South America, including Brazil (53). The seropositivity to ZIKV (22.3%) was much lower than previous reports (57%–63.3%) during the ZIKV outbreak (2015−2017) in Northeastern Brazil using the same Euroimmun kit (54, 55). It is worth noting that the samples in our study were collected between 2021 and 2022, ~6 years after the peak of ZIKV outbreak in the region; a previous study showed decline of ZIKV seroprevalence over time, from 24% (2015) to 12% (2017), in French Polynesia and Fiji, including 23 (12.2%) seroreversions among 189 paired sera (56). Similarly, a rapid decline of anti-ZIKV NS1 IgG was reported in Northeastern Brazil from 59.0% (2015) to 38.6% (1.5–2 years later), with a high (20.6%) seroreversion rate (57). The relatively weak anti-ZIKV NS1 antibody response compared with the anti-E antibody response may account for such a decline, and this should be considered for future ZIKV seroprevalence studies (15, 16, 31). A recent systematic review revealed heterogeneity of global ZIKV seroprevalence with a seroprevalence rate of 39.9% (95% CI: 30.0%–49.9%) in the Americas (58). In line with this, ZIKV seroprevalence was reported to be 34.6% (range: 23.4%–47.4%) at Recife in Northeastern Brazil (2018−2019) using the Euroimmun kit (59)

The seropositive rates of DENV (70.3%) and CHIKV (5.7%) were comparable to those of DENV (75.7%; CI: 69.4%–81.1%) and CHIKV (7.4%; CI: 5.6%–9.8%) reported previously in Northeastern Brazil (54). Notably, the pattern of the age-stratified seropositivity of DENV, ZIKV, and CHIKV was generally in agreement with that based on the Euroimmun kits (Fig. 4I and 5E). An abrupt increase in DENV seropositivity was observed between age group <19 and 20–39, followed by a peak in age groups 20–39 and 40–59, and slight decline in age groups 60–79 and ≥80, which was consistent with a recent report in Northeast Brazil (Table S4) (59). An increase in ZIKV seropositivity was also observed between age groups <19 and 20–39, followed by a gradual decline with age, raising the possibility of age-related decline of anti-ZIKV NS1 IgG over time. Consistent with previous reports, the seropositivity of DENV and CHIKV was higher in females than males, but not significant for CHIKV (Table S4) (59, 60). For CHIKV, a recent study reported a seroprevalence rate of 22.1% (range: 2.0%–70.5%) at Feira de Santana in Northeastern Brazil (60); our CHIKV seropositive rate is within the range. The high heterogeneity of CHIKV seropositivity in intra-urban space was in line with that of CHIKV seroprevalence from six studies in Brazil (7.4%–51%) and between countries and territories (13.1%–57.9%) as reported in a recent systematic review (13, 61). Given the low sensitivity (52.2%) of anti-YFV NS1 in IgG MIA, further analysis of our YF-17D vaccinees panel revealed that the anti-YFV NS1 detection rate was higher among those with evidence of other orthoflavivirus infection/exposure (2/2 = 100%) based on history and WB analysis compared with those without (5/12 = 41.6%), albeit the small sample size. With a DENV seropositivity of 70.3% in Saúde, ~70% of YFV seropositive participants who had previous DENV infection will be detected by DENV WT VLP, and also detected by anti-YFV NS1 at a higher rate (close to 100%) or interpreted as unspecified orthoflavivirus infection and verified by WB analysis, whereas ~30% of YFV seropositive participants who had no previous DENV infection will be detected by anti-YFV NS1 at a rate of 41.6%, suggesting approximately 17.5% YFV infection/exposure might be missed in Saúde. Future studies should include YFV VLP together with DENV1 WT VLP as the pan-orthoflavivirus marker to capture all YFV infections/exposure. Nonetheless, the seropositivity of YFV (39.7%) was within the range of the estimated coverage rate of YFV vaccination in Brazil (30%–70% for individuals aged 15–70 years) and suggested they had previous YFV vaccination or infection (62). As current serological tests cannot distinguish YFV vaccination and natural infection, the possibility of YFV infection during the recent or previous outbreaks in Brazil cannot be ruled out (8).

There are several limitations. First, the sample size in Saúde (*n* = 300) is small; nonetheless, the sampling rate (2.86%, 300/10,478 population of Saúde) is high compared with other seroprevalence studies (34, 54, 55, 59, 60). Second, future studies involving a sufficient number of sequential samples with different orthoflavivirus infections, especially those with repeated orthoflavivirus infections such as DENV + ZIKV, DENV + YFV, or DENV + ZIKV + YFV, are needed to better assess the performance of the IgG MIA over time. Third, although historically flaviviruses were separated into different serocomplexes using hyperimmune animal sera in NT (63), extensive cross-reactivity was observed in E-protein-based binding assays (14–17). We chose to employ eight panels of human serum samples with well-documented arbovirus infections or vaccination, which represent antibody profiles in humans, to evaluate the sensitivity/specificity of NS1 and E-protein-based antigens in our multiplex IgG MIA. Notably, while the magnitude of antibody responses to WNV is thought to be lower in asymptomatic blood donors compared with neuroinvasive WNV cases (64–66), the cutoff rMFI in our WNV-NS1 IgG MIA based on blood donors is likely to increase the sensitivity of detecting previous WNV infection. The possible decrease in specificity will be validated by WB or NT analysis as proposed in our algorithm. Fourth, due to the lack of serum samples from confirmed MAYV cases or MAYV-specific human monoclonal antibodies, MAYV antigen was not included in our IgG MIA for alphavirus detection and should be included in future studies. Nonetheless, pseudovirus NT to both CHIKV and MAYV is recommended for equivocal samples and confirmation of CHIKV infection in the proposed algorithm of this study (Fig. 3D, Fig. S2). Lastly, future studies to distinguish other pathogenic arboviruses such as JEV, tick-borne encephalitis virus (TBEV), Onyongnyong, Ross River, and Oropouche viruses, depending on the endemicity in different regions, remain to be exploited. In light of the successful implementation of several orthoflavivirus vaccines and ongoing vaccine trials in endemic regions, serological tests that can distinguish natural infection and vaccinations with DENV, JEV, YFV, and TBEV vaccines are warranted (2, 3, 6).

In summary, we reported a multiplex IgG MIA for five pathogenic arboviruses with validated sensitivity/specificity using eight panels of samples with well-documented arbovirus infection or vaccination and showed that the majority of study participants from a town in Northeastern Brazil had multiple arbovirus infections or exposure, including DENV + ZIKV, DENV + YFV, DENV + ZIKV + YFV, and/or CHIKV. This high-throughput and multiplex assay, combined with confirmatory tests, provides a convenient and reliable tool to explore serodiagnosis and serosurveillance of medically important arboviruses in endemic regions.

## Data Availability

The data presented in this study are available from the corresponding author upon request.

## AFTER EPUB

*[This article was published on 30 April 2026 with an error in an affiliation. The affiliation was corrected in the current version, posted on 11 May 2026.]*

## SUPPLEMENTAL MATERIAL

Supplemental tables and figures jcm.00013-26-s0001.pdf Tables S1 to S4 and Figures S1 to S6.

ASM does not own the copyrights to Supplemental Material that may be linked to, or accessed through, an article. The authors have granted ASM a non-exclusive, world-wide license to publish the Supplemental Material files. Please contact the corresponding author directly for reuse.
