# Supplementary material for: Identification of multiple arbovirus infections/exposure in Northeastern Brazil using a multiplex microsphere immunoassay
Source: J Clin Microbiol. 2026 Apr 30;64(6):e00013-26. doi: 10.1128/jcm.00013-26 (PMC13251372; doi:10.1128/jcm.00013-26)
Supplement: Supplemental tables and figures — Tables S1 to S4 and Figures S1 to S6. [file jcm.00013-26-s0001.pdf]

TABLE S1 Numbers, sources and basic information of different serum/plasma panels with confirmed arbovirus infections or vaccination

Panel[a]	No. of samples	Sampling time[b]	Sources [No. of samples], Country and year (reference)	Confirmation methods
pDENV	40	6M−31Y	seroprevalence study [12], Taiwan, 2015−16 (32)	NT[c]
		1M−5.6Y	DENV study [11], Taiwan, 2001−9 (20)	RT-PCR[d]
		2.7M−10M	DENV study [11], Hawaii, 2015 (20)	RT-PCR[d]
		1M−1.5Y	DENV study [6], Nicaragua, 2006−8 (20)	RT-PCR[d]
sDENV	111	1M−30Y	seroprevalence study [24], Taiwan, 2015−16 (32)	NT[c]
		<1M−6Y	DENV study [55], Taiwan, 2001−9 (20)	RT-PCR[d]
		3M−5.6M	DENV study [2], Hawaii, 2015 (20)	RT-PCR[d]
		3M−1Y	DENV study [30], Nicaragua, 2006−8 (20)	RT-PCR[d]
pZIKV	12	1.5M−1.3Y	ZIKV study [12], Brazil, 2016−17 (31)	NT[c]
DENV+ZIKV	22	3M-2.3Y	ZIKV study [22], Brazil, 2016−17 (31)	NT[c]
YFV-17D	23	<1M−5Y	YFV-17D study [10], U.S. 1990−2016 (30)	vaccination history
		unknown	ZIKV study [9], Brazil, 2016−17 (31)	vaccination history
		1M−1.5Y	NHP study [4], BEI Resources, 2011−12	vaccination history
WNV	18	<1M	WNV study [18], U.S. 2006−15 (20)	TMA[e]
CHIKV	22	2M	CHIKV study [22], Brazil, 2016−18 (34)	RT-PCR[d]
DENV-negative	135	NA	seroprevalence study [135], Taiwan, 2015−16 (32)	NT[c] or multiple ELISAs

[a]pDENV, primary DENV infection; sDENV, secondary DENV infection; WNV, WNV infection; pZIKV, primary ZIKV infection; DENV+ZIKV, previous DENV and ZIKV infections; YFV-17D, YFV-17D vaccination; NHP, non-human primates; NA, not applicable.
[b]Sampling time post-symptom onset, YFV-17D vaccination, or TMA test.
[c]Microneutralization test (NT) and history based on questionnaires in the study as described previously (31-33).
[d]Reverse transcription-polymerase chain reaction (RT-PCR) and history as described previously (20); no enough sample for NT.
[e]Index samples tested positive for WNV transcription-mediated amplification (TMA), IgM and IgG from blood donors at the American Red Cross (20), no enough sample for NT.

TABLE S2 Comparing the sensitivity and specificity of different DENV NS1 IgG MIA

Viral antigen[c]	Group	% Sensitivity (95% CI)[a,b]	% Specificity (95% CI)[a,b]
D1−4 NS1	overall	96.0 (93.0–97.5)	98.3 (96.4–99.3)
	subgroup	pDENV:90.0, sDENV:97.3, DENV+ZIKV:100	DENV-negative:99.3, pZIKV:100, WNV:94.4, YF-17D:92.9
D1 NS1	overall	96.0 (93.0–97.5)	96.7 (94.0–98.0)
	subgroup	pDENV:82.5, sDENV:100, DENV+ZIKV:100	DENV-negative:99.3, pZIKV:83.3, WNV:94.4, YF-17D:85.7
D2 NS1	overall	96.0 (93.0–97.5)	97.2 (94.8–98.4)
	subgroup	pDENV:90.0, sDENV:97.3, DENV+ZIKV:100	DENV-negative:99.3, pZIKV:100, WNV:88.9, YF-17D:85.7
D3 NS1	overall	93.6 (90.0–95.5)	96.1 (93.3–97.5)
	subgroup	pDENV:82.5, sDENV:96.4, DENV+ZIKV:100	DENV-negative:99.3, pZIKV:83.3, WNV:88.9, YF-17D:85.7
D4 NS1	overall	93.1 (89.3–95.0)	96.7 (94.0–98.0)
	subgroup	pDENV:85.0, sDENV:94.6, DENV+ZIKV:100	DENV-negative:97.8, pZIKV:91.7 WNV:94.4, YF-17D:92.9

[a]CI, confidence interval. pDENV, primary DENV infection; sDENV, secondary DENV infection; pZIKV, primary ZIKV infection; DENV+ZIKV, previous DENV and ZIKV infections; WNV, WNV infection; YF-17D, YF-17D vaccination;
[b]For simplicity, the 95% CIs in the subgroup are not shown.
[c]NS1, nonstructural protein 1; VLP, virus-like particles; D1, DENV1; D2, DENV2; D3, DENV3, D4, DENV4.

TABLE S3 Comparing the sensitivity and specificity of different DENV VLP and E IgG MIA

Viral antigen[c]	Group	% Sensitivity (95% CI)[a,b]	% Specificity (95% CI)[a,b]
D1 WT VLP	overall	100 (100–100)	82.1 (76.5–85.0)
	subgroup	pDENV:100, sDENV:100, DENV+ZIKV:100	DENV-negative:100, pZIKV:16.7, WNV:0, YF-17D:71.4
D1 FL VLP	overall	100 (100–100)	86.0 (81.0–88.6)
	subgroup	pDENV:100, sDENV:100, DENV+ZIKV:100	DENV-negative:100, pZIKV:50.0, WNV:16.7, YF-17D:71.4
D1 E	overall	98.3 (96.3–99.3)	82.1 (76.5–85.0)
	subgroup	pDENV:95.0, sDENV:99.1, DENV+ZIKV:100	DENV-negative:98.5, pZIKV:33.3, WNV:0, YF-17D:85.7

[a]CI, confidence interval. pDENV, primary DENV infection; sDENV, secondary DENV infection; pZIKV, primary ZIKV infection; DENV+ZIKV, previous DENV and ZIKV infections; WNV, WNV infection; YF-17D, YF-17D vaccination.
[b]For simplicity, the 95% CIs in the subgroup are not shown.
[c]VLP, virus-like particles; E, envelope protein; WT, wild type; FL, fusion loop-mutant; D1, DENV1.

TABLE S4 Basic information of study participants compared to study population in Saúde and seroprevalence to DENV, ZIKV, YFV and CHIKV among different age groups and gender

Groups[a]	Saude population[b] No. (%)	Study participants No. (%)	Seroprevalence[c] No. of positive/samples tested (%)			
			DENV	ZIKV	YFV	CHIKV
total	10,478 (100.0%)	300 (100.0%)	211/300 (70.3%)	67/300 (22.3%)	119/300 (39.7%)	17/300 (5.7%)
Age group						
≤19	3,011 (28.7%)	22 (7.3%)	11/22 (50.0%)	0/22 (0%)	8/22 (36.4%)	0/22 (0%)
20−39	2,662 (25.4%)	123 (41.0%)	95/123[d] (77.2%)	38/123[d] (30.9%)	57/123 (46.3%)	10/123 (8.1%)
40−59	2,785 (26.6%)	103 (34.3%)	78/103 (75.7%)	22/103 (21.4%)	38/103 (36.9%)	7/103 (6.8%)
60−79	1,654 (15.8%)	43 (14.3%)	21/43 (48.8%)	6/43 (14.0%)	15/4 (34.9%)	0/43 (0%)
≥80	366 (3.5%)	9 (3.0%)	6/9 (66.7%)	1/9 (11.1%)	1/9 (11.1%)	0/9 (0%)
Gender						
male	5,147 (49.1%)	166 (55.3%)	108/166 (65.1%)	40/166 (24.1%)	64/166 (38.6%)	8/166 (4.8%)
female	5,331 (50.9%)	134 (44.7%)	103/134[e] (76.9%)	27/134 (20.1%)	55/134 (41.0%)	9/134 (6.7%)

[a]Basic information includes total numbers, age groups and gender of study population in Saúde and study participants.
[b]Information based on Census data in 2022.
[c]Based on IgG MIA combined with confirmatory tests.
[d]Comparing age groups ≤19 and 20−39, $P<0.01$, Fisher's exact test.
[e]Comparing male and female, $P=0.03$, Fisher's exact test.

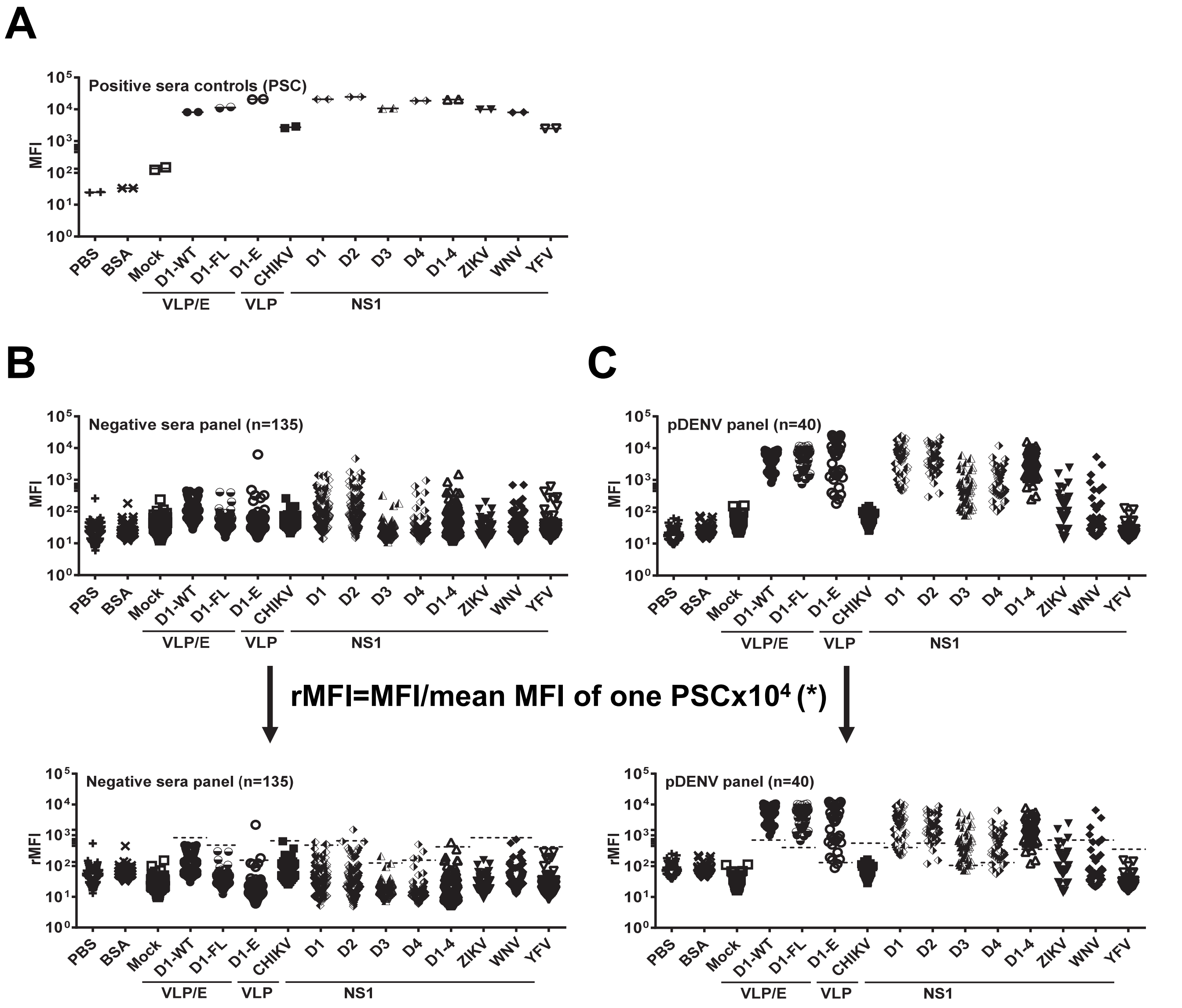

FIG S1 Determination of rMFI and cutoff rMFI. (A) Positive sera controls, (B) Negative sera panel, and (C) pDENV panel. The MFI values for each antigen were divided by the mean MFI value of one positive sera control (MFI~10^4) and multiplied by 10^4 (*by 100 and 3000 for negative antigen controls and CHIKV VLP, respectively) to calculate rMFI. The cutoff rMFI for each antigen was defined by the mean rMFI value of 20 negative sera controls (from DENV-negative panel) plus 5 standard deviations. Data are the means (each in duplicate), and dashed lines indicate cutoff rMFI.

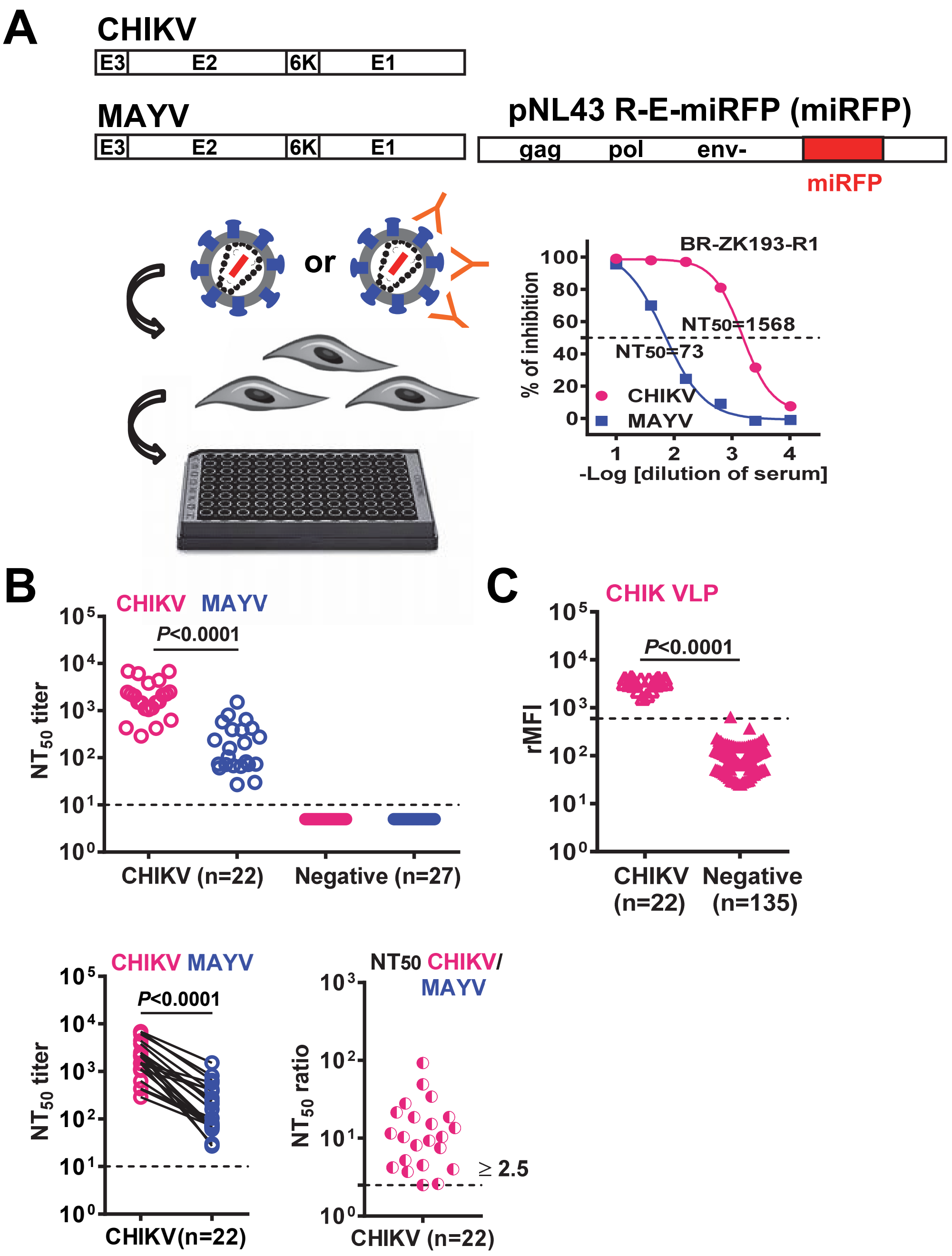

FIG S2 CHIKV and MAYV pseudovirus NTs and IgG MIA of CHIKV VLP.
(A) Schematic drawing of plasmids expressing E2 and E1 proteins (E3-E2-6K-E1 genes) of CHIKV or MAYV and co-transfection with pNL43 R-E-miRFP (miRFP) to generate CHIKV or MAYV pseudoviruses containing miRFP reporter, and neutralization curves with NT_{50} titers to CHIKV and MAYV at 72 h post-infection in Huh7 cells using convalescent-phase serum sample from an RT-PCT-confirmed CHIKV case. (B) NT_{50} titers to CHIKV and MAYV pseudoviruses in RT-PCR confirmed CHIKV (n=22) and negative (n=27) sera panels, and ratios of NT_{50} titers of CHIKV to MAYV (≥2.5 in CHIKV panel, lower right)[38-40]. (C) rMIF to CHIKV VLP in CHIKV (n=22) and negative (n=135) sera panels. The two-tailed Wilcoxon signed-rank test and Mann-Whitney test were performed in panels B and C, respectively.

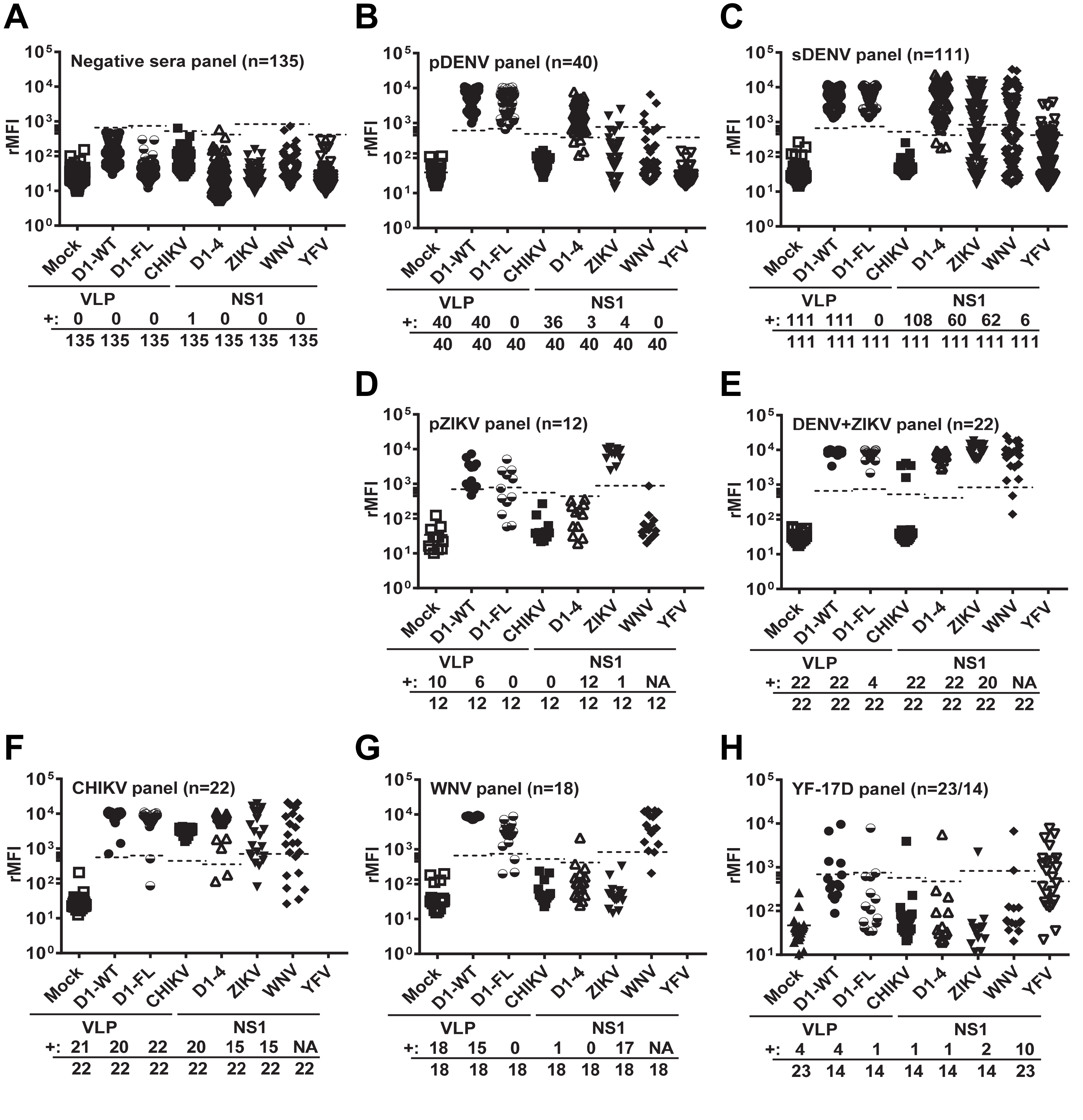

FIG S3 Results of the multiplex IgG MIA tested with 8 panels of samples with known arbovirus infection/exposure. Results of IgG MIA for 8 panels of serum/plasma samples: negative sera (A), pDENV (B), sDENV (C), pZIKV (D), DENV+ZIKV (E), CHIKV (F), WNV (G), and YF-17D vaccination (H) panels. The 7 down-selected antigens and one negative antigen control include NS1 (DENV1−4, ZIKV, WNV, and YFV) and VLP (mock, DENV1 WT, DENV1 FL, and CHIKV). Numbers of positive and total samples tested for each panel are shown below each graph. NA, not applicable due to lack of history of YFV infection or vaccination in some panels. Data are the means (each in duplicate), and dashed lines indicate cutoff rMFI.

FS4

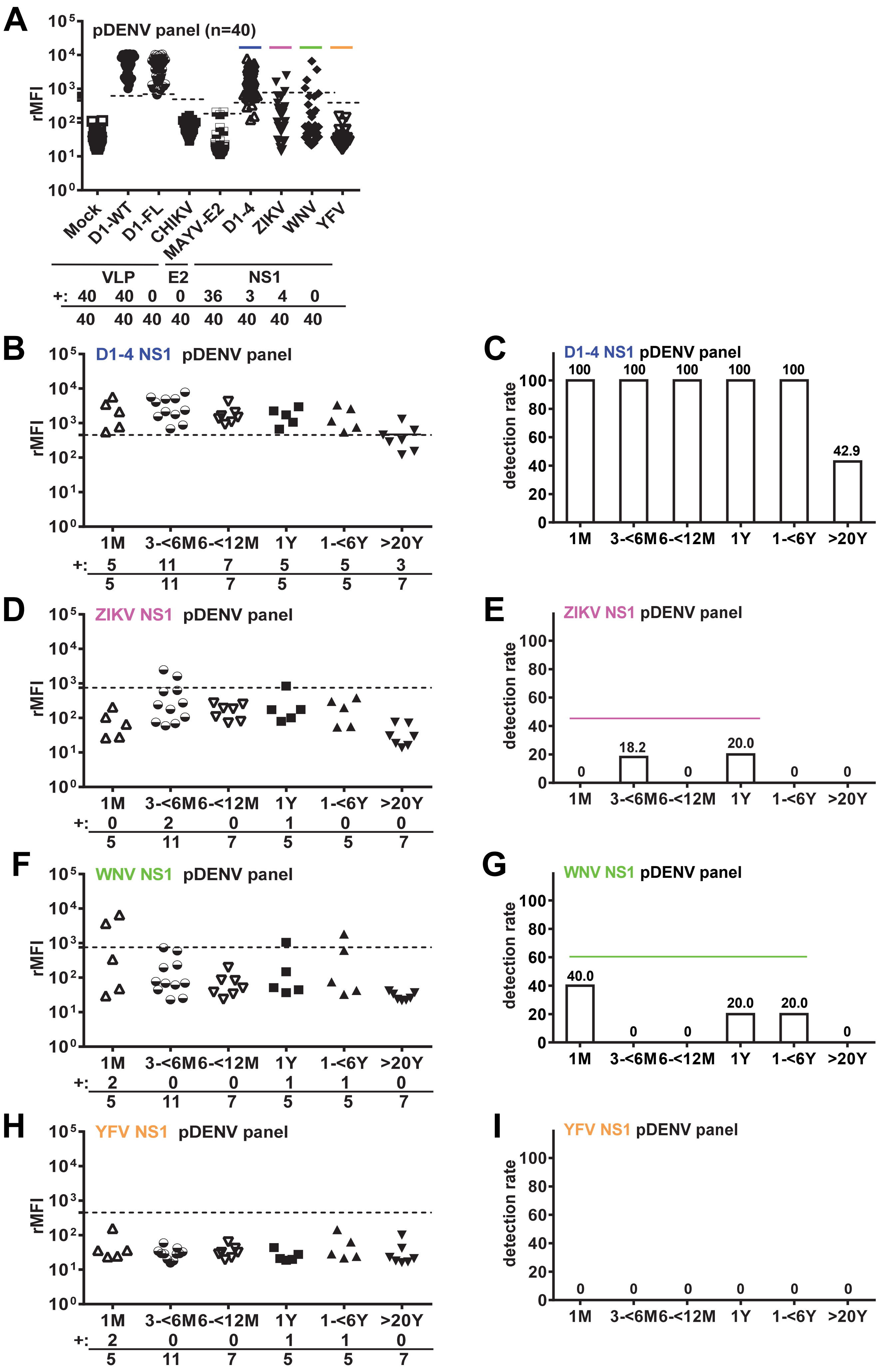

A
pDENV panel (n=40)
rMFI
Mock
D1-WT
D1-FL
CHIKV
MAYV-E2
D1-4
ZIKV
WNV
YFV
VLP
E2
NS1
+: 40 40 0 0 36 3 4 0
40 40 40 40 40 40 40 40
B
D1-4 NS1 pDENV panel
1M 3-<6M 6-<12M 1Y 1-<6Y >20Y
+: 5 11 7 5 5 3
5 11 7 5 5 7
C
D1-4 NS1 pDENV panel
detection rate
100 100 100 100 100 42.9
D
ZIKV NS1 pDENV panel
+: 0 2 0 1 0 0
5 11 7 5 5 7
E
ZIKV NS1 pDENV panel
0 18.2 0 20.0 0 0
F
WNV NS1 pDENV panel
+: 2 0 0 1 1 0
5 11 7 5 5 7
G
WNV NS1 pDENV panel
40.0 0 0 20.0 20.0 0
H
YFV NS1 pDENV panel
+: 2 0 0 1 1 0
5 11 7 5 5 7
I
YFV NS1 pDENV panel
0 0 0 0 0 0

FIG S4 Results of NS1 IgG MIA and cross-reactivities over time after pDENV infection. Results of multiplex IgG MIA in pDENV panel (A), and NS1 IgG MIA and detection rate over time, including DENV1−4 (B,C), ZIKV (D,E), WNV (F,G), and YFV (H,I) NS1 proteins. Numbers of positive and total samples tested for each subgroup are shown below each graph with rMFI. Cross-reactivities (%) to ZIKV NS1 within 1 year and to WNV NS1 within 6 years are highlighted with lines (E,G). Data are the means (each in duplicate), and dashed lines indicate cutoff rMFI.

A

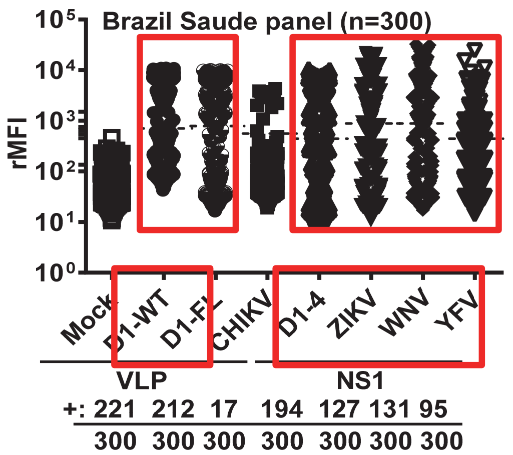

B

	D	ZK	YF	D only	ZK only	D+ ZK	YF only	D+ YF	D+ ZK+ YF	neg
n	193	66	96	95	4	19	17	36	43	66
%	64.3	22.0	32.0	31.7	1.3	6.3	5.7	12.0	14.3	22.0

	possible W?	unspecified Ortho flavivirus
n	15	20
%	5.0	6.7

C

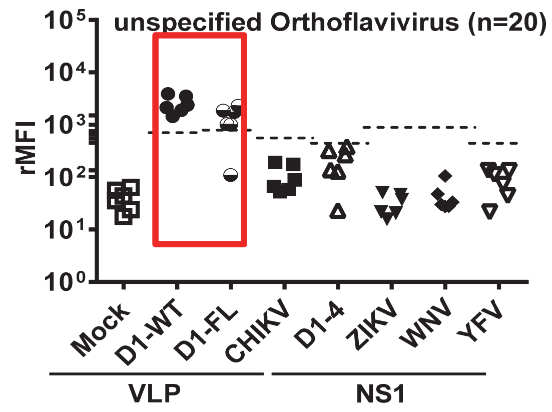

D

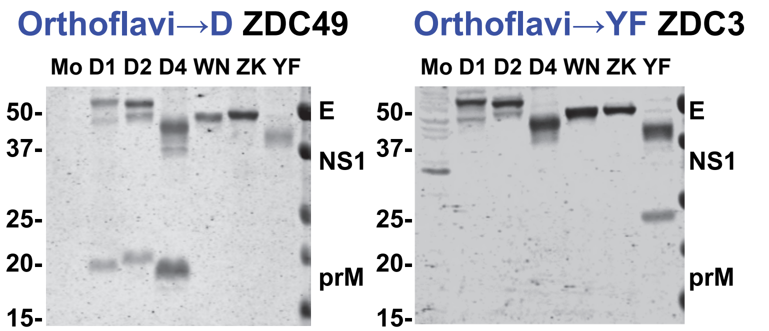

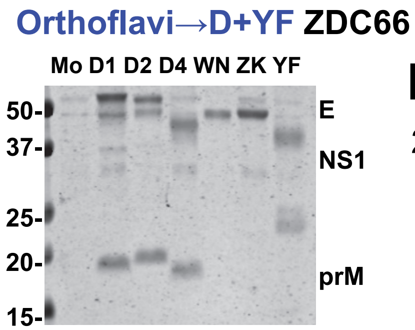

E

20 unspecified → 7 D
Orthoflavi 1 YF
12 D+YF

F

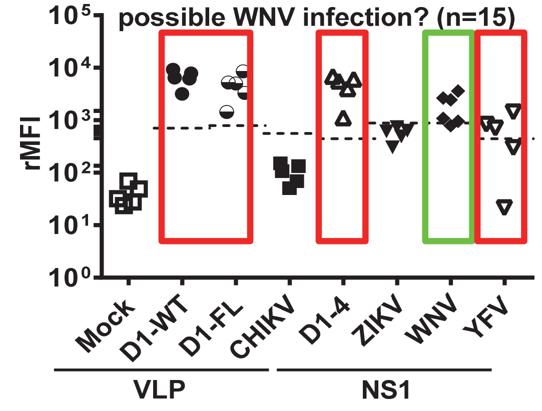

G

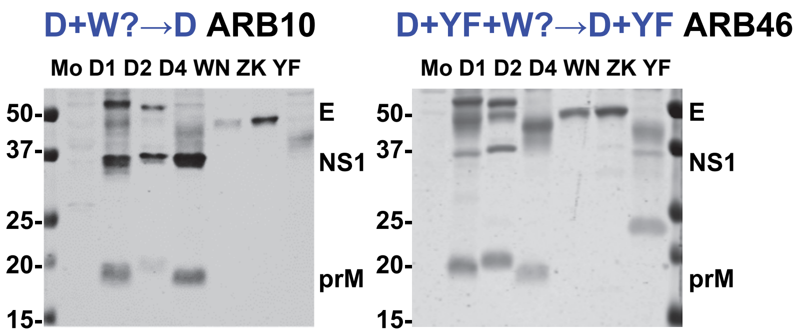

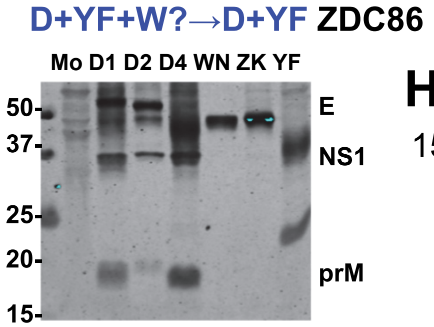

H

15 possible W? → 3 D
(D+W? or 1 YF
D+YF+W?) 11 D+YF

FIG S5 Confirmation of seropositivity of orthoflaviviruses in Saúde. (A,B) Results of multiplex IgG MIA tested for serum samples collected from Saude with orthoflaviviruses highlighted (A) and numbers of positive for one or multiple orthoflavivirus infections and total samples tested (B), including 20 unspecified orthoflavivirus infections and 15 possible WNV infections. Numbers of positive and total samples tested for each antigen are shown below panel A. (C-E) Results of multiplex IgG MIA of 20 samples of unspecified orthoflavivirus infections based on positivity to DENV1 WT VLP and negative to all NS1 tested (C), representative Western blot gels for identifying specific orthoflavivirus infections (D), and summary of 20 identified orthoflavivirus infections (E). (F-H) Results of multiplex IgG MIA of 15 samples with possible WNV infection based on positivity to WNV NS1 protein (F), representative Western blot gels for confirming specific orthoflavivirus infections (G), and summary of 15 confirmed orthoflavivirus infections (H).

FS6 revised

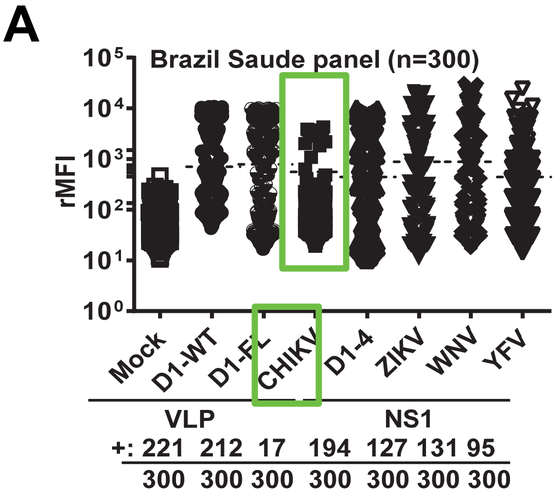

B

CHIKV VLP	n	total (n=300)
+	17	CHIKV
−	283	negative

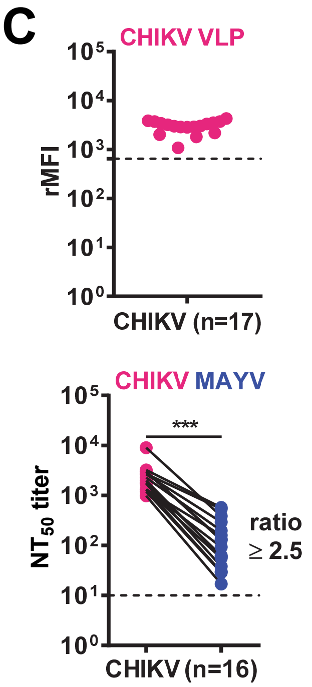

FIG S6 Confirmation of seropositivity of CHIKV in Saúde. (A,B) Results of multiplex IgG MIA tested for serum samples collected from Saude with CHIKV highlighted (A) and numbers of positivity for CHIKV (B). Numbers of positive and total samples tested for each antigen are shown below panel A. (C) rMFI of CHIKV VLP (upper) and NT_{50} titers with ratios of NT_{50} titers of CHIKV to MAYV pseudovirus (lower) for 17 CHIKV infections.
